# Supplementary material for: Charting the Chemical Reaction Space around a Multicomponent Combination: Controlled Access to a Diverse Set of Biologically Relevant Scaffolds
Source: Angew Chem Int Ed Engl. 2023 Jun 2;62(41):e202303889. doi: 10.1002/anie.202303889 (PMC10952796; doi:10.1002/anie.202303889)

checkCIF/PLATON report

Structure factors have been supplied for datablock(s) 1_a

THIS REPORT IS FOR GUIDANCE ONLY. IF USED AS PART OF A REVIEW PROCEDURE FOR PUBLICATION, IT SHOULD NOT REPLACE THE EXPERTISE OF AN EXPERIENCED CRYSTALLOGRAPHIC REFEREE.

No syntax errors found.   CIF dictionary   Interpreting this report

Datablock: 1_a

```
Bond precision:  C-C = 0.0072 A          Wavelength=1.54178

Cell:           a=15.815(2)    b=7.4619(10)        c=12.7516(16)
                alpha=90       beta=103.016(9)     gamma=90
Temperature:    180 K

                Calculated                 Reported
Volume          1466.2(3)                  1466.2(3)
Space group     P 21/c                     P 1 21/c 1
Hall group      -P 2ybc                    -P 2ybc
Moiety formula  C34 H26 Cl2 N4 O2          C34 H26 Cl2 N4 O2
Sum formula     C34 H26 Cl2 N4 O2          C34 H26 Cl2 N4 O2
Mr              593.49                     593.49
Dx,g cm-3       1.344                      1.344
Z               2                          2
Mu (mm-1)       2.299                      2.299
F000            616.0                      616.0
F000'           619.04
h,k,lmax        17,8,14                    17,8,14
Nref            2191                       2182
Tmin,Tmax       0.802,0.977                0.538,0.752
Tmin'           0.795

Correction method= # Reported T Limits: Tmin=0.538 Tmax=0.752
AbsCorr = MULTI-SCAN

Data completeness= 0.996          Theta(max)= 60.091

R(reflections)= 0.0652( 1356)     wR2(reflections)= 0.2252( 2182)

S = 1.098                  Npar= 190
```

The following ALERTS were generated. Each ALERT has the format
test-name_ALERT_alert-type_alert-level.
Click on the hyperlinks for more details of the test.

Alert level B

THETM01_ALERT_3_B The value of sine(theta_max)/wavelength is less than 0.575
Calculated sin(theta_max)/wavelength = 0.5622

Author Response: The best possible small crystal has been sought and used for data collection.It has been measured with low temperature and Cu radiation in order to improve the reflection intensities, but the crystal gives weakly diffractions which has not been detectable above theta values.

Alert level C

```
PLAT242_ALERT_2_C Low    'MainMol' Ueq as Compared to Neighbors of         C6 Check
PLAT242_ALERT_2_C Low    'MainMol' Ueq as Compared to Neighbors of        C15 Check
PLAT340_ALERT_3_C Low Bond Precision on  C-C Bonds ...............    0.00718 Ang.
PLAT905_ALERT_3_C Negative K value in the Analysis of Variance ...     -7.187 Report
PLAT905_ALERT_3_C Negative K value in the Analysis of Variance ...     -0.662 Report
PLAT911_ALERT_3_C Missing FCF Refl Between Thmin & STh/L=    0.562         10 Report
```

Alert level G

```
PLAT072_ALERT_2_G SHELXL First  Parameter in WGHT  Unusually Large       0.11 Report
PLAT913_ALERT_3_G Missing # of Very Strong Reflections in FCF ....          2 Note
PLAT978_ALERT_2_G Number C-C Bonds with Positive Residual Density.          0 Info
```

0 **ALERT level A** = Most likely a serious problem - resolve or explain
1 **ALERT level B** = A potentially serious problem, consider carefully
6 **ALERT level C** = Check. Ensure it is not caused by an omission or oversight
3 **ALERT level G** = General information/check it is not something unexpected

0 ALERT type 1 CIF construction/syntax error, inconsistent or missing data
4 ALERT type 2 Indicator that the structure model may be wrong or deficient
6 ALERT type 3 Indicator that the structure quality may be low
0 ALERT type 4 Improvement, methodology, query or suggestion
0 ALERT type 5 Informative message, check

It is advisable to attempt to resolve as many as possible of the alerts in all categories. Often the minor alerts point to easily fixed oversights, errors and omissions in your CIF or refinement strategy, so attention to these fine details can be worthwhile. In order to resolve some of the more serious problems it may be necessary to carry out additional measurements or structure refinements. However, the purpose of your study may justify the reported deviations and the more serious of these should normally be commented upon in the discussion or experimental section of a paper or in the "special_details" fields of the CIF. checkCIF was carefully designed to identify outliers and unusual parameters, but every test has its limitations and alerts that are not important in a particular case may appear. Conversely, the absence of alerts does not guarantee there are no aspects of the results needing attention. It is up to the individual to critically assess their own results and, if necessary, seek expert advice.

Publication of your CIF in IUCr journals

A basic structural check has been run on your CIF. These basic checks will be run on all CIFs submitted for publication in IUCr journals (*Acta Crystallographica*, *Journal of Applied Crystallography*, *Journal of Synchrotron Radiation*); however, if you intend to submit to *Acta Crystallographica Section C* or *E* or *IUCrData*, you should make sure that full publication checks are run on the final version of your CIF prior to submission.

Publication of your CIF in other journals

Please refer to the *Notes for Authors* of the relevant journal for any special instructions relating to CIF submission.

PLATON version of 05/12/2020; check.def file version of 05/12/2020

Datablock 1_a - ellipsoid plot

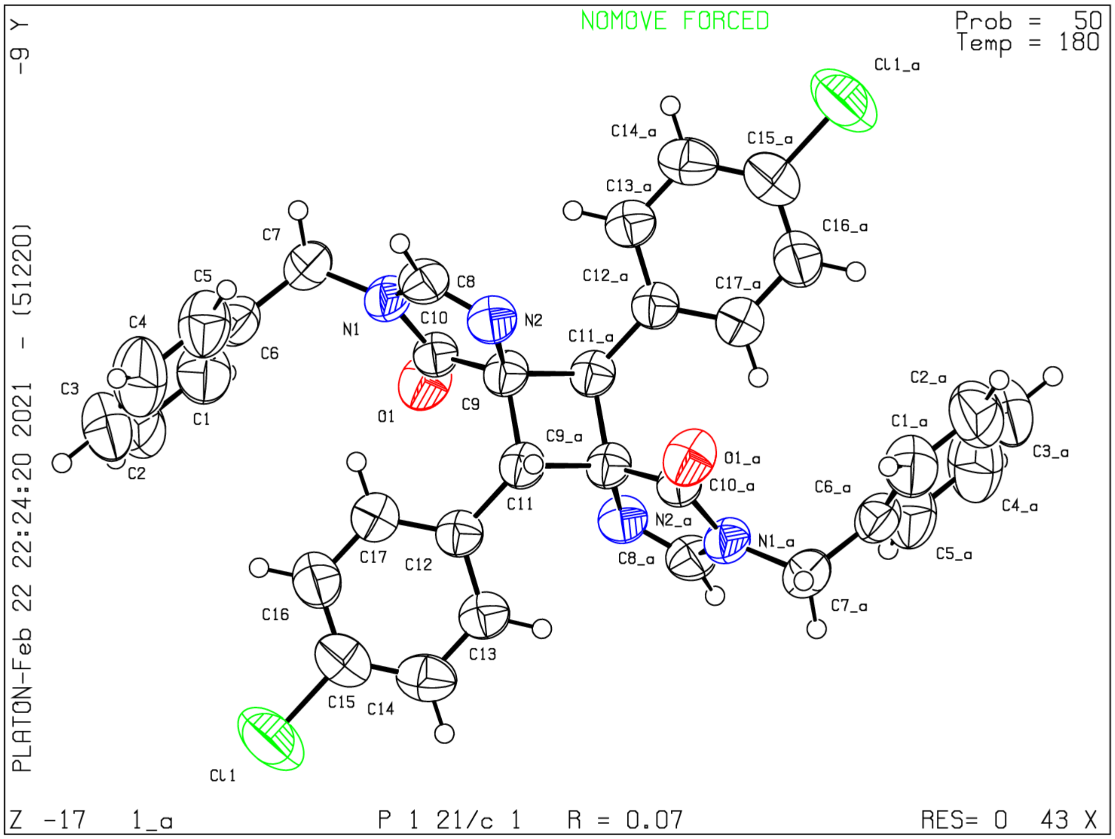

Supplement: Supplementary file 1 [file ANIE-62-0-s001.pdf]
